# Systems pharmacology and drug repositioning- an integrated approach to metabolic diseases

**DOI:** 10.1186/1479-5876-10-S2-A18

**Published:** 2012-10-17

**Authors:** Michael A Luther

**Affiliations:** 1 The David H Murdock Research Institute; Kannapolis, North Carolina, USA

## 

The discovery of new therapeutics is highly risky endeavor. Current estimates show the average timeline from early discovery to market is greater than twelve years at a cost of over $1 billion per each new NCE. The lack of translation from pre-clinic to clinic has been identified as a major bottleneck. This has led to the question of how we can improve the probability of success in drug discovery and development. One approach is to use a systems pharmacology approach to provide a comprehensive phenotype approach to disease and potential therapeutics vs. the reductionist approach which was a dominant approach the last 20 years. This comprehensive phenotypic approach or, systems pharmacology, can also be applied to phenol typing marketed and failed therapeutics as well as to therapeutics progressing from candidate selection to Phase II. Statistics show that from 2007-09, 30-40% of drugs or biologics that were approved or launched for the first time in the US were either drugs repositioned for new indications, reformulations or new combinations of existing drugs. The identification of new indications for failed or approved therapeutics has been referred to as drug repurposing or drug repositioning. Historically, this repositioning has come from serendipitous discoveries in late stage clinical trials or post-approval. Examples include Viagra, Thalidomide, and Glevec. Using an integrated approach through the application of systems biology technologies for in vitro and in vivo pharmacology studies provides the opportunity to move drug repositioning to earlier stages in drug development and apply a more deterministic vs. serendipitous approach to identify new indications from late stage lead discovery to Phase II studies. This talk focuses on strategies, tactics, and techniques employed to develop a systems pharmacology approach to identify and validate new indications. Examples will be provided demonstrating success at the lead, candidate, and failed assets as well as potential new indications for marketed therapeutics.

